# Obesity increases neuropathic pain via the AMPK-ERK-NOX4 pathway in rats

**DOI:** 10.18632/aging.203305

**Published:** 2021-07-29

**Authors:** Chang-Ning Fu, Hui Wei, Wen-Shuang Gao, Sha-Sha Song, Shou-Wei Yue, Yu-Juan Qu

**Affiliations:** 1 Rehabilitation Center, Qilu Hospital, Cheelo College of Medicine, Shandong University, Jinan, China; 2 Department of Critical Care Medicine, Shandong Provincial Hospital Affiliated to Shandong First Medical University, Jinan, China

**Keywords:** extracellular-regulated kinase, obesity, neuropathic pain

## Abstract

This study focused on the relationship between extracellular-regulated kinase (ERK) and obesity-induced increases in neuropathic pain. We fed rats a high-fat diet to establish the obesity model, and rats were given surgery to establish the chronic compression of the dorsal root ganglia (CCD) model. U0126 was applied to inhibit ERK, and metformin or 5-aminoimidazole-4-carboxamide ribonucleoside (AICAR) was applied to cause AMP-activated protein kinase (AMPK) activation. Paw withdrawal mechanical threshold (PWMT) were calculated to indicate the level of neuropathic pain. The data indicated that compared with normal CCD rats, the PWMT of obese CCD rats were decreased, accompanied with an increase of ERK phosphorylation, NAD(P)H oxidase 4 (NOX4) protein expression, oxidative stress and inflammatory level in the L4 to L5 spinal cord and dorsal root ganglia (DRG). Administration of U0126 could partially elevate the PWMT and reduce the protein expression of NOX4 and the above pathological changes in obese CCD rats. *In vitro*, ERK phosphorylation, NOX4 protein expression increased significantly in DRG neurons under the stimulation of palmitic acid (PA), accompanied with increased secretion of inflammatory factors, oxidative stress and apoptosis level, while U0126 partially attenuated the PA-induced upregulation of NOX4 and other pathological changes. In the rescue experiment, overexpression of NOX4 abolished the above protective effect of U0126 on DRG neurons in high-fat environment. Next, we explore upstream mechanisms. Metformin gavage significantly reduced neuropathic pain in obese CCD rats. For the mechanisms, activating AMPK with metformin (obese CCD rats) or AICAR (DRG neurons in a high-fat environment) not only inhibited the ERK-NOX4 pathway, but also improved oxidative stress and inflammation caused by high-fat. In conclusion, the AMPK-ERK-NOX4 pathway may has a pivotal role in mediating obesity-induced increases in neuropathic pain.

## INTRODUCTION

Obesity and pain are two serious public health problems in our society. Recent studies have shown that obesity has a close relationship with increased neuropathic pain [1–3], including chronic pain [4]. However, the molecular mechanisms underlying neuropathic pain induced by obesity have not been thoroughly elaborated.

As a pivotal signaling system, the mitogen-activated protein kinase (MAPK) family are widely existing, and at least four MAPK pathways have been identified: the extracellular-related kinases (ERK1/2), ERK5, p38-MAPK and Jun amino-terminal kinases (JNK1/2/3). According to the study, the MAPK/ERK pathway was involved in many cellular functions, including migration, differentiation, apoptosis, and senescence [5]. More importantly, the MAPK/ERK pathway has a pivotal role in many neurodegenerative diseases [6], and targeting the ERK signaling pathway is an innovative therapeutic strategy for treating neurodegeneration [7]. Our previous study has reported that the ERK pathway is an important mechanism in neuropathic pain in rats with a chronic compression of dorsal root ganglia (CCD) model, and targeting ERK with U0126 or lentivirus expressing ERK shRNA significantly alleviates chronic compression of dorsal root ganglia (DRG)-induced neuropathic pain [8, 9].

Atsuko Matoba et al. found that elevated plasma free fatty acids levels promote cell proliferation and p70S6K phosphorylation by activating MEK/ERK pathway in obese individuals [10]. Shangyu Hong et al. reported that the ERK pathway is abnormally activated in adipocytes [11]. Is the increased neuropathic pain induced by obesity related to the abnormality of the ERK signaling pathway in nerve tissue? Based on the above studies, we hypothesized that under obese conditions, abnormal activation of the ERK signaling pathways in the nervous tissue leads to increased neuropathic pain in rats. Moreover, the upstream and downstream molecular mechanisms of ERK should also be explored.

In this study, we demonstrated that in the DRG and spinal cord of obese CCD rats, the ERK signaling pathway is excessively activated and an ERK inhibitor alleviates the increased neuropathic pain induced by obesity. The ERK inhibitor may have a beneficial effect by decreasing the content of NAD(P)H oxidase 4 (NOX4) in nervous tissue. While for the upstream mechanism, the ERK signaling pathway activation may result from inactivation of the AMP-activated protein kinase (AMPK). Clinically, targeting the AMPK-ERK-NOX4 pathway may be an effective strategy to treat obesity-induced hyperalgesia in the future.

## MATERIALS AND METHODS

### CCD model and experimental protocols

Male Wistar rats (150-180 g) were purchased from the Shandong University Animal Center. The rats were free to take food and water with three per cage.

We fed the rats in the obese group a high-fat diet (20% sucrose, 15% axunge, and 5% cholesterol) for 12 weeks to establish an obesity model, and a normal diet was applied for the control group rats. If the average body weight of rats in the obese group was 20% higher compared with rats in the control group, the obese model was successfully established.

Both normal and obese rats underwent CCD surgery. We established the CCD model according to our previous methods [12]. After anesthesia, the L4 and L5 intervertebral foramen were inserted unilaterally with two stainless steel rods. Rats exhibiting autophagy, feeling deficiency, and disability were excluded.

**Figure 1:**
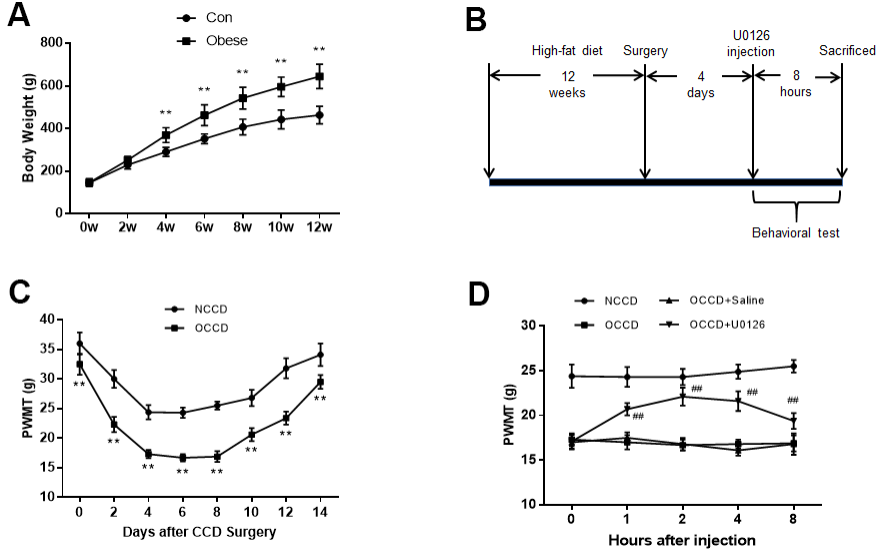
**Inhibition of ERK activation reduces obesity-induced increases in neuropathic pain.** (**A**) The body weight of rats was measured at different time points. (**B**) The protocol of Part 1. (**C**) Allodynia in the NCCD and OCCD groups was detected by the PWMT test at the indicated time points after CCD surgery. (**D**) The role of ERK inhibition on PWMT in obese CCD rats after intrathecal injection. N = 10-15 per group. **P < 0.01 vs. con or NCCD group, ^##^*P* < 0.01 vs. OCCD + saline group. U0126, ERK inhibitor; ERK, extracellular-regulated kinase; AMPK, AMP-activated protein kinase; CCD, chronic compression of the dorsal root ganglia; NCCD, normal CCD; OCCD, obese CCD; PWMT, paw withdrawal mechanical threshold.

**Figure 2:**
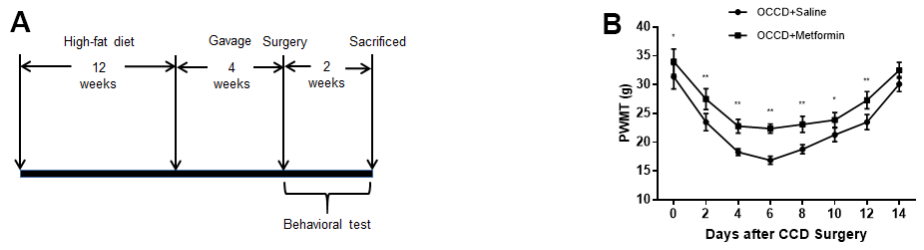
**AMPK activation attenuates neuropathic pain enhancement in obese rats.** (**A**) The protocol of Part 2. (**B**) Allodynia in the OCCD+saline and OCCD+metformin groups was detected by the PWMT test at the indicated time points after CCD surgery.. N = 10-15 per group, ^*^P < 0.05 vs. OCCD+saline group, ^**^P < 0.01 vs. OCCD+saline group. OCCD, obese CCD; PWMT, paw withdrawal mechanical threshold.

Animal experiments were divided into two parts. Part 1: To verify the effect of inhibiting ERK activation on neuropathic pain in obese CCD rats and explore the underlying mechanism. The experiment was divided into four groups: NCCD group (normal rats with surgery), OCCD group (obese rats with surgery), saline group (OCCD rats with saline injection) and U0126 group (OCCD rats with U0126 injection). 15 rats in each group, a total of 60 rats. At the end of the experiment, 3 rats died in OCCD group, 5 rats died in saline group and 3 rats died in U0126 group. As shown in Figure 1B, CCD surgery was performed at week 12 of the experimental period. Intrathecal injections of U0126 (ERK inhibitor, CST, Boston, USA, concentration = 40 M) [9] were given 4 days after CCD surgery in obese rats, and behavioral tests were performed before and 1, 2, 4, and 8 h after injection. Then, the rats were sacrificed. Part 2: To verify the effect of activating AMPK on neuropathic pain and ERK signaling pathway in obese rats. The experiment was divided into four groups: NCCD group (normal rats with surgery), OCCD group (obese rats with surgery), saline group (OCCD rats with saline gavage) and metformin group (OCCD rats with metformin gavage). 15 rats in each group, a total of 60 rats. At the end of the experiment, 2 rats died in NCCD group, 5 rats died in OCCD group, 4 rats died in saline group and 2 rats died in metformin group. As shown in Figure 2A, the experimental group rats were given a high-fat diet followed by metformin (or saline) intragastric administration for the next 4 weeks (300 mg·kg^-1^ of metformin per day) [13]. After CCD surgery, behavioral tests were performed daily for 14 days, after which the rats were sacrificed. The L4 and L5 ganglia and dorsal horn of spinal cord from the operated side were harvested.

The Animal Care and Use Committee of Shandong University Qilu Hospital reviewed and approved all the experimental protocols. The ethical approval reference number is DWLL-2018-021.

### Behavioral testing of paw withdrawal mechanical thresholds

The test method is based on our previous reports [8]. The rats were fully accustomed to the test environment before the test. On the testing day, we placed the rats in their individual Plexiglas enclosures to acclimatize for 30 min before testing. BME-404 Mechanical Analgesia Tester (CAMS-Chinese Academy of Medical Sciences, Beijing, China) was used to evaluate the paw withdrawal mechanical threshold (PWMT). We followed the method of a previous study [9] for testing. The interval between the two tests was more than 5 minutes, and the test was repeated 5 times for each rat. Average values were calculated.

### DRG neuron cultures and treatment

After the rats were anesthetized, L4 and L5 ganglia were quickly isolated, cut into pieces, and digested with collagenase and trypsin at 37° C for 30 min. We collected DRG neurons referring to a previous method [14]. According to the instructions, the DRG neurons were stimulated with U0126 (CST, concentration = 10 μM) or 5-aminoimidazole-4-carboxamide ribonucleoside (AICAR) (CST, concentration = 2 mM) for 2 h, and then received other stimuli. The high-fat environment of cells was simulated by palmitic acid (PA) (TargetMol, USA, concentration = 0.4 mM) stimulation as previously reported [15].

### Western blot analysis

After cells or tissues were lysed, we used a BCA Protein Assay Kit (Boster Bio, USA) to determine the protein concentration. Total protein was separated by SDS-PAGE. We used a constant current to transfer the separated protein to a PVDF membrane. 5% skim milk was applied to block non-specific reaction sites for 1-2 h. Dilute the primary antibody at the concentration recommended by the instructions, immerse the membrane in this liquid, and place at 4° C for at least 8 hours. The primary antibodies: anti-GAPDH (Proteintech, Wuhan, China), anti-p-ERK (CST), anti-ERK (CST), anti-NOX4 (Abcam, Cambridge, UK), anti-p-AMPK (Abcam), anti-AMPK (Abcam). Horseradish peroxidase-conjugated secondary antibodies were diluted at the concentrations recommended by the instructions, and the membranes were incubated for 2h at room temperature. Enhanced chemiluminescence reagent was used to visualize the protein bands in an imaging densitometer (GE, USA).

### RNA extraction and real-time polymerase chain reaction

According to the RNA Extraction Kit (Fastgen, Shanghai, China) instructions, Total RNA was obtained by tissue lysis and centrifugation. The cDNA is synthesized by a Prime Script 1st Strand cDNA Synthesis Kit (Takara, Kyoto City, Japan). Real-time quantitative polymerase chain reaction (PCR) was applied to measure the interest gene levels. Reaction mixtures had a volume of 20 μL and contained SYBR® Premix Ex Taq™ II (Takara). Reactions were run for 40 cycles with specified temperature change. The target gene values of each sample were averaged and normalized to the *U6* gene. The primer sequences are as follows: Interleukin-1 beta (IL-1β), 5′-ACCTTCCAGGATGAGGACATGA-3′ and 5′-CACACACCAGCAGGTTA-3′; Interleukin-6 (IL-6), 5′-AGTTGCCTTCTTGGGACTGA-3′ and 5′-TCCACGATTTCCCAGAGAAC-3′; Tumor necrosis factor alpha (TNF-α), 5′-AAGCAAGCAGCCAACCAG-3′ and 5′-TCTTCTGCCAGTTCCACG-3′.

### Immunohistochemistry

Tissue wrapped in wax blocks were sectioned into tissue sections, then dewaxed with dewaxing solution and hydrated with gradient alcohol. After that, the sections were placed in antigen repair solution at a temperature of >95° C for 15 min to expose antigen. In order to block non-specific reactions, 10% goat serum was dripped onto the tissue surface. The primary antibody was diluted at the recommended concentration, dripped onto the tissue surface, incubated with sections for at least 8 hours at 4° C, and complete coverage is guaranteed. Diluted horseradish peroxidase-labeled secondary antibody was applied. The color was developed with DAB chromogen at room temperature, and the time depended on the color change. The nuclei were slightly stained with hematoxylin for several seconds, and the tissues were dehydrated, transparently treated, and sealed. A digital imaging system (Olympus Corporation, Tokyo, Japan) was applied to capture images. Densitometry were applied for calculations and statistics.

### Malondialdehyde, superoxide dismutase and NADPH oxidase activity assay

The kits (NanJing JianCheng Bioengineering Institute, Nanjing, China) were applied to determine Superoxide dismutase (SOD), Malondialdehyde (MDA), and NADPH oxidase activity. The manufacturer's instructions were strictly followed by the determination procedures.

### Measurement of reactive oxygen species content

The treated cells were washed three times and then incubated with oxidant-sensitive fluorescence probe dihydroethidium (DHE) (Beyotime Biotechnology, Beijing, China) at a concentration of 5 μM for 30 minutes. Excitation/emission wavelengths of 535/610 nm were used to capture images (Olympus, Japan).

### Enzyme-linked immunosorbent assay

After stimulating the DRG neurons with PA solution for enough time, the supernatants in each group were collected and used to detect the secretion of IL-1β, IL-6, and TNF-α. The Enzyme-linked Immunosorbent Assay Kit (ELISA Kit) instructions (RayBio, USA) were strictly followed by the determination procedures.

### TUNEL assay

Apoptotic levels of DRG neurons were determined by a TUNEL Assay Kit (Beyotime Biotechnology, Beijing, China). Excitation/emission wavelengths of 488/520 nm were applied to capture images (Olympus, Japan).

### Blood glucose, total cholesterol, and triglycerides tests

Rat blood glucose was measured using a glucometer (Roche), and blood total cholesterol (TC) and triglycerides (TG) were determined by a Total Cholesterol Assay Kit and a Triglyceride Assay Kit (Nan Jing Jian Cheng Bioengineering Institute, Nanjing, China). All test procedures were according to the manufacturers’ protocols.

### Statistical analysis

All quantitative data are expressed as mean ± standard deviation. Normality of these data were tested before analyzing. We used GraphPad Prism 8 or SPSS20.0 to analysis all the data. Unpaired Student's t-test was applied for two groups comparison. One-way analysis of variance (ANOVA) was applied for more groups. The comparison was considered statistically significant when the two-sided *P* < 0.05.

## RESULTS

### Basic characteristics of rats

Relevant data are shown in Table 1.

**Table 1:** Basic characteristics of rats.

**Characteristic**	**NCCD**	**OCCD**	**Saline (intrathecal)**	**U0126**	**Saline (gavage)**	**Metformin**
Weight (g)	478.8±48.4	663.4±62.1**	660.4±58.3	667.8±65.3	668.2±59.5	652.3±60.2
Blood Glucose (mmol/L)	6.8±1.6	10.4±2.3*	10.8±1.8	10.5±2.1	9.8±1.7	7.3±1.4^#^
TC (mmol/L)	1.8±0.8	6.8±2.5**	6.7±2.3	6.5±2.8	6.2±2.1	6.2±1.9
TG (mmol/L)	1.1±0.3	3.2±1.3**	3.2±1.1	3.4±1.5	3.0±1.1	2.9±1.3

*P<0.05 or **P<0.01 vs. NCCD group, ^#^*P*<0.05 vs. saline (gavage) group.

The determination methods are shown in the Materials and Methods.

### Inhibition of ERK activation can reduce obesity-induced increases in neuropathic pain

High-fat-fed rats gained body weight rapidly compared with control group rats. The obese group rats began to weigh significantly more than the control group rats from the 4th week of feeding. At the 12th week, the weight of obese group rats was 39.2% higher than that in the control group, which was more than 20% of the set standard (Figure 1A). The obesity model was successfully established.

Figure 1B shows the procedure of rats receiving high-fat diet feeding, undergoing CCD surgery, receiving intrathecal injection of U0126, and undergoing behavioral testing.

As shown in Figure 1C, after CCD surgery, the decrease in PWMT in obese rats (OCCD group) was more obvious than that in normal rats (NCCD group), which indicated that obese rats had higher neuropathic pain.

To examine the effect of inhibiting ERK activation on obesity-induced neuropathic pain elevation, we administered U0126 intrathecally to obese rats on the fourth day after CCD surgery. PWMT were measured multiple times before and within 8 h after injection. Compared with the OCCD group, the intrathecal saline injection did not significantly affect the PWMT of obese CCD rats, but PWMT were increased significantly from 1 h after intrathecal U0126 injection, peaked at 2 h, and there was still a statistically significant increase at 8 h (Figure 1D). This suggests that inhibition of ERK activation can attenuate the increased allodynia induced by obesity in rats.

### ERK phosphorylation, NOX4 expression, oxidative stress and inflammation are elevated in the L4-L5 spinal cord and DRG of obese CCD rats, and decreased by an ERK inhibitor

**Figure 3:**
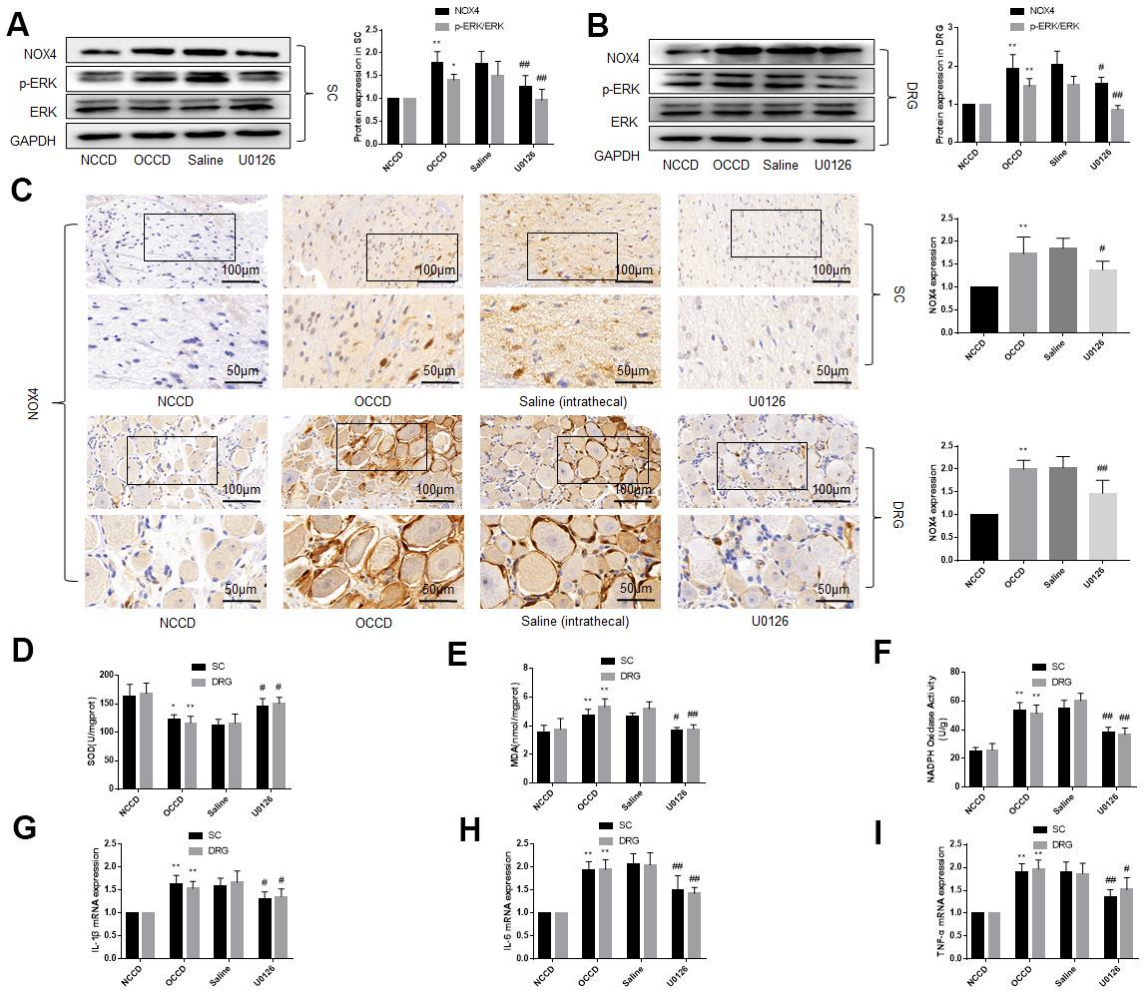
**ERK phosphorylation, NOX4 expression, oxidative stress and inflammation are elevated in the L4, L5 spinal cord and DRG of obese CCD rats, which are abolished by an ERK inhibitor.** (**A**–**C**) The protein levels of p-ERK, ERK, and NOX4 in nervous tissue of rats were determined by western blot and immunohistochemistry. (**D**–**F**) SOD, MDA, NADPH oxidase activity in nervous tissue were detected by kit. (**G**–**I**) IL-1β, IL-6, and TNF-α mRNA levels in nervous tissue were detected by PCR. N = 10-15 per group, *P < 0.05 vs. NCCD group, **P < 0.01 vs. NCCD group, ^#^*P* < 0.05 vs. saline group, ^##^*P* < 0.01 vs. saline group. U0126, ERK inhibitor; ERK, extracellular-regulated kinase; NOX4, NAD(P)H oxidase 4; DRG, dorsal root ganglia; CCD, chronic compression of the dorsal root ganglia; MDA, malondialdehyde; SOD, superoxide dismutase; IL-1β, interleukin-1 beta; IL-6, interleukin-6; TNF-α, tumor necrosis factor alpha; qRT-PCR, quantitative real-time polymerase chain reaction; NCCD, normal CCD; OCCD, obese CCD.

After the behavioral tests in Part 1, the rats were sacrificed, and their L4-L5 spinal cord and DRG tissues were isolated. Western blotting and immunohistochemistry showed that ERK phosphorylation and NOX4 protein levels in the DRG and spinal cord were higher in the OCCD group compared the NCCD group, and intrathecal saline injection could not change their expression, but U0126 significantly decreased the ERK phosphorylation and NOX4 expression (Figure 3A–3C).

We examined the oxidative stress and inflammation of the L4-L5 spinal cord and DRG by measuring SOD et al. by kit and the levels of IL-1β, IL-6, and TNF-α mRNA by PCR. The results demonstrated the oxidative stress and inflammation of nervous tissue in the OCCD group was more severe than that in the NCCD group, but the intrathecal injection of U0126, instead of saline, attenuated these pathological changes (Figure 3D–3I). These data suggest that inhibiting the activation of ERK in the nervous tissue of obese rats can reduce the oxidative stress and inflammatory response caused by obesity.

### An ERK inhibitor reduced NOX4 expression, and improves inflammation, oxidative stress and apoptotic level of DRG neurons cultured in PA-added medium *in vitro*

**Figure 4:**
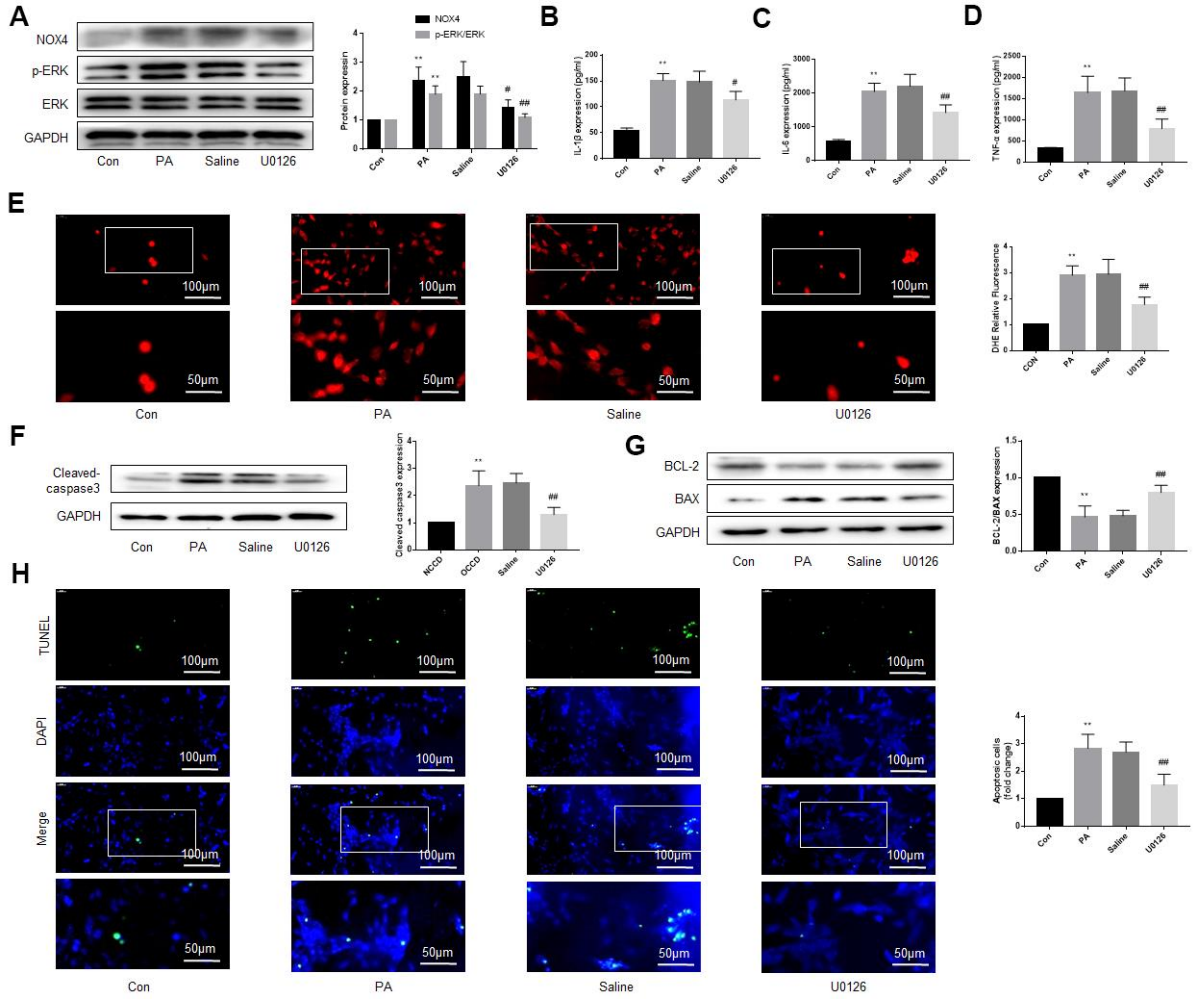
**An ERK inhibitor improves NOX4 expression, inflammation, oxidative stress and apoptotic level of DRG neurons cultured in PA-added medium *in vitro*.** PA was applied to simulate a high-fat environment and U0126 to inhibit ERK. (**A**) The expression of p-ERK, ERK, and NOX4 in DRG neurons were detected by western blot. (**B**–**D**) The supernatant of cultured cells was collected and the secretion of IL-1β, IL-6, and TNF-α was determined by ELISA. (**E**) The ROS content was detected by oxidant-sensitive fluorescence probe DHE. (**F**, **G**) The expression of cleaved caspase3, bcl-2 and bax in DRG neurons were detected by western blot. (**H**) Cell apoptosis level was detected by TUNEL method. N = 5 per group, **P < 0.01 vs. con group, #*P* < 0.05 vs. saline group, ##*P* < 0.01 vs. saline group. U0126, ERK inhibitor; ERK, extracellular-regulated kinase; NOX4, NAD(P)H oxidase 4; PA, palmitic acid; DRG, dorsal root ganglia; IL-1β, interleukin-1 beta; IL-6, interleukin-6; TNF-α, tumor necrosis factor alpha; ELISA, enzyme-linked immunosorbent assay; DHE, dihydroethidium; ROS, reactive oxygen species.

We added PA to the culture medium of DRG neurons to simulate a high-fat environment. The results indicated that ERK phosphorylation, NOX4 protein levels, secretion of inflammatory factors, oxidative stress level of the DRG neurons were significantly increased under the stimulation of PA. U0126 treatment, but not saline treatment, decreased the ERK phosphorylation, NOX4 expression, inflammatory and the oxidative stress (Figure 4A–4E).

We also used western blot and TUNEL to detect the apoptosis of DRG neurons under high-fat environment and the effect of inhibiting ERK activation on apoptosis. As shown in Figure 4F, 4G, under the stimulation of PA, the expression of cleaved caspase3 and bax increased obviously, but the expression of bcl-2, an anti-apoptotic protein, decreased. U0126 alleviated the above effects of PA, but saline had no similar effect. TUNEL assay was consistent with the above results (Figure 4H).

These results suggest that the ERK inhibitor protects DRG neurons from damage caused by high fat. Considering the close relationship between NOX4 and inflammation, oxidative stress and apoptosis [16–19], the protective effect of the ERK inhibitor may be related to the reduction of NOX4.

### Overexpression of NOX4 abolishes the protective effect of an ERK inhibitor on DRG cells under high-fat environment

**Figure 5:**
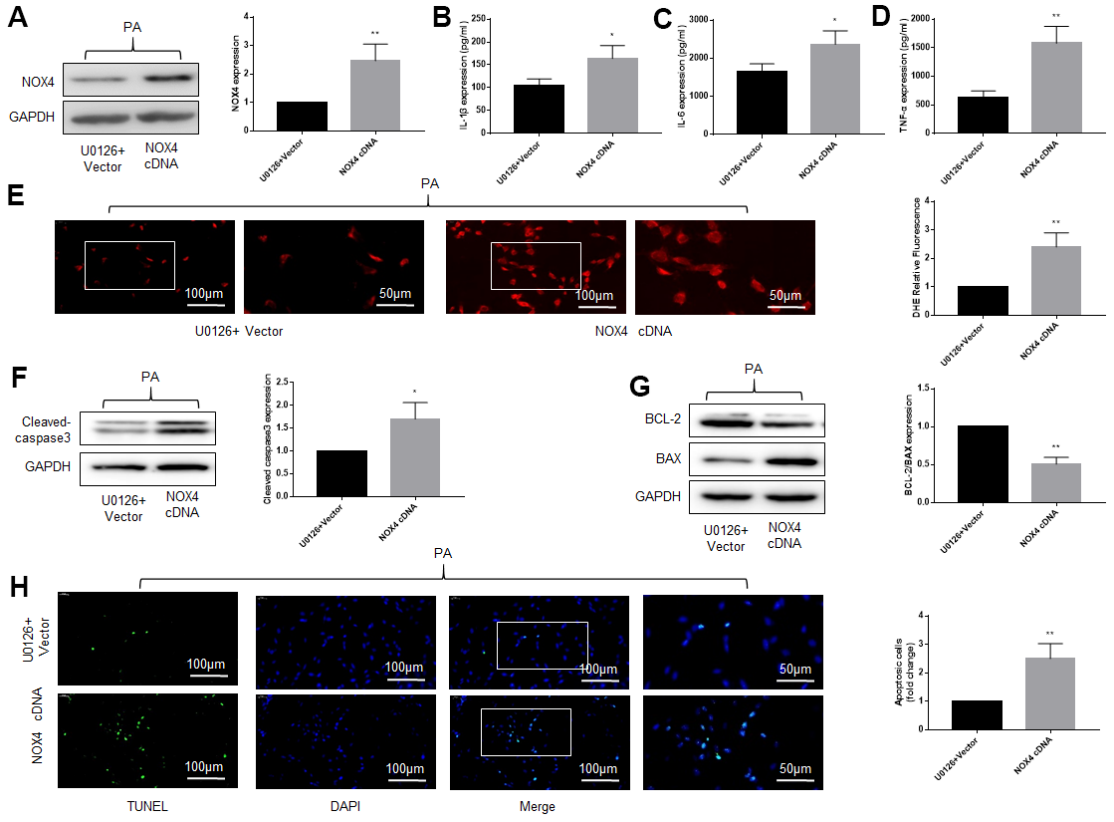
**Overexpression of NOX4 abolishes the protective effect of an ERK inhibitor on DRG cells under high-fat environment.** DRG neurons isolated from rats were cultured. PA was applied to simulate a high-fat environment and U0126 to inhibit ERK. NOX4 cDNA was transfected into cells to overexpress NOX4. (**A**) The transfection effect of NOX4 was indicated by western blot. (**B**–**D**) The supernatant of cultured cells was collected and the secretion of IL-1β, IL-6, and TNF-α was determined by ELISA. (**E**) The ROS content was detected by oxidant-sensitive fluorescence probe DHE. (**F**, **G**) The expression of cleaved caspase3, bcl-2 and bax in DRG neurons were detected by western blot. (**H**) Cell apoptosis level was detected by TUNEL method. N = 5 per group, *P < 0.05 vs. U0126+Vector group, **P < 0.01 vs. U0126+Vector group. U0126, ERK inhibitor; ERK, extracellular-regulated kinase; NOX4, NAD(P)H oxidase 4; PA, palmitic acid; DRG, dorsal root ganglia; IL-1β, interleukin-1 beta; IL-6, interleukin-6; TNF-α, tumor necrosis factor alpha; ELISA, enzyme-linked immunosorbent assay; DHE, dihydroethidium; ROS, reactive oxygen species.

Next, we explored whether the protective effect of U0126 on neural tissue under high-fat environment was NOX4-dependent. The DRG neurons were first stimulated with PA and U0126, and then the NOX4 cDNA was transfected into the DRG neurons. After NOX4 cDNA were transferred into cells, the expression of NOX4 protein increased significantly (Figure 5A). As shown in Figure 5B–5H, overexpression of NOX4 increased inflammation, oxidative stress and apoptosis of DRG neurons compared with U0126+Vector group (Figure 5B–5H). This indicates that the increase of NOX4 protein abolishes the protective effect of U0126 on DRG neurons under high-fat environment, that is, the protective effect of the ERK inhibitor on cells is NOX4-dependent.

### Promotion of AMPK activation can reduce obesity-induced increases in neuropathic pain

Figure 2A reveals the procedure of rats receiving high fat-diet feeding, undergoing CCD surgery, receiving intragastric administration, and undergoing behavioral testing.

To explore the function of activating AMPK on neuropathic pain in obese rats, we administered metformin intragastrically to obese rats for 4 weeks, followed by CCD surgery and behavioral tests. The results showed that compared with intragastric administration of saline, metformin significantly increased PWMT in obese CCD rats, suggesting that activation of AMPK in obese rats reduced allodynia (Figure 2B).

### Activation of AMPK in obese CCD rats reduces the ERK phosphorylation, NOX4 expression and attenuates the oxidative stress and inflammatory in the L4-L5 spinal cord and DRG

**Figure 6:**
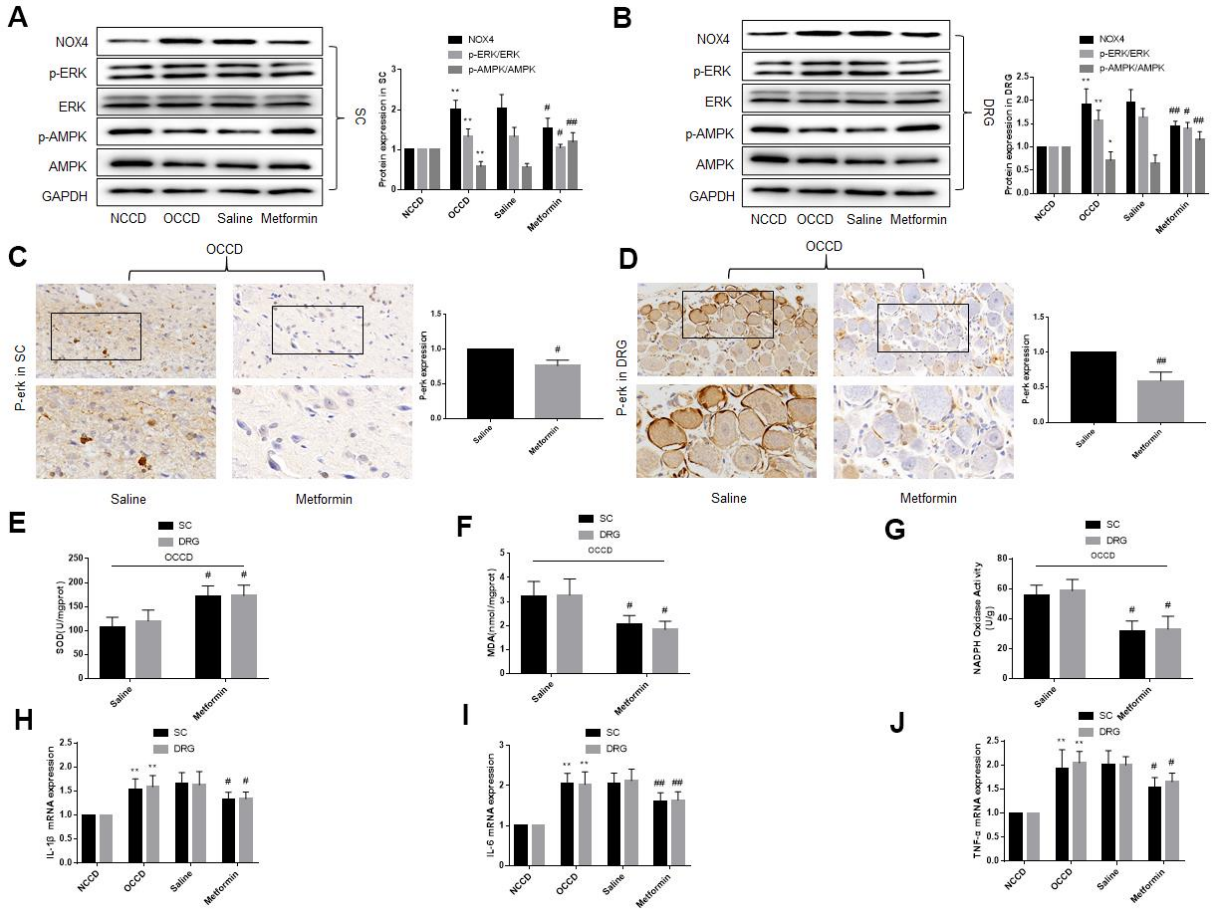
**AMPK activation in obese CCD rats reduces the ERK phosphorylation, NOX4 expression, oxidative stress and inflammatory response in L4, L5 spinal cord and DRG of obese CCD rats.** (**A**–**D**) The expression of p-AMPK, AMPK, p-ERK, ERK, and NOX4 in nervous tissue of rats were determined by western blot and immunohistochemistry. (**E**–**G**) SOD, MDA, NADPH oxidase activity in the nervous tissue were detected by kit. (**H**–**J**) IL-1β, IL-6, and TNF-α mRNA levels in nervous tissue were detected by PCR. N = 10-15 per group, *P < 0.05 vs. NCCD group, **P < 0.01 vs. NCCD group, ^#^*P* < 0.05 vs. saline group, ^##^*P* < 0.01 vs. saline group. AMPK, AMP-activated protein kinase; U0126, ERK inhibitor; ERK, extracellular-regulated kinase; NOX4, NAD(P)H oxidase 4; DRG, dorsal root ganglia; CCD, chronic compression of the dorsal root ganglia; MDA, malondialdehyde; SOD, superoxide dismutase; IL-1β, interleukin-1 beta; IL-6, interleukin-6; TNF-α, tumor necrosis factor alpha; qRT-PCR, quantitative real-time polymerase chain reaction; NCCD, normal CCD; OCCD, obese CCD.

On the basis of previous studies, AMPK has a relation with pain [20–23], so we speculated that in the nervous tissue of obese rats, abnormal of AMPK may lead to the increase of ERK phosphorylation and NOX4 expression, thereby inducing the increased neuropathic pain in obese rats. After the behavioral test was completed in Part 2, the L4 to L5 spinal cord and DRG were isolated and the levels of AMPK, ERK, and NOX4 expression were detected. AMPK phosphorylation in the L4 to L5 spinal cord and DRG from the OCCD group was lower compared with NCCD group, while ERK phosphorylation and NOX4 expression were higher. Intragastric administration of metformin, but not saline, significantly increased AMPK phosphorylation, but decreased ERK phosphorylation and NOX4 expression (Figure 6A–6D).

Compared with NCCD group, expression of IL-1β, IL-6, and TNF-α mRNA, content of SOD, MDA and NADPH oxidase in the OCCD group increased. Not intragastric administration of saline, but metformin significantly reduced these pathological changes (Figure 6E–6J).

These data suggest that p-AMPK may be the upstream molecule of p-ERK and NOX4 in the nervous tissue of obese CCD rats.

### AMPK phosphorylation reduces ERK phosphorylation and NOX4 expression and thus improves the pathological status of DRG neurons cultured in PA-supplemented medium *in vitro*

**Figure 7:**
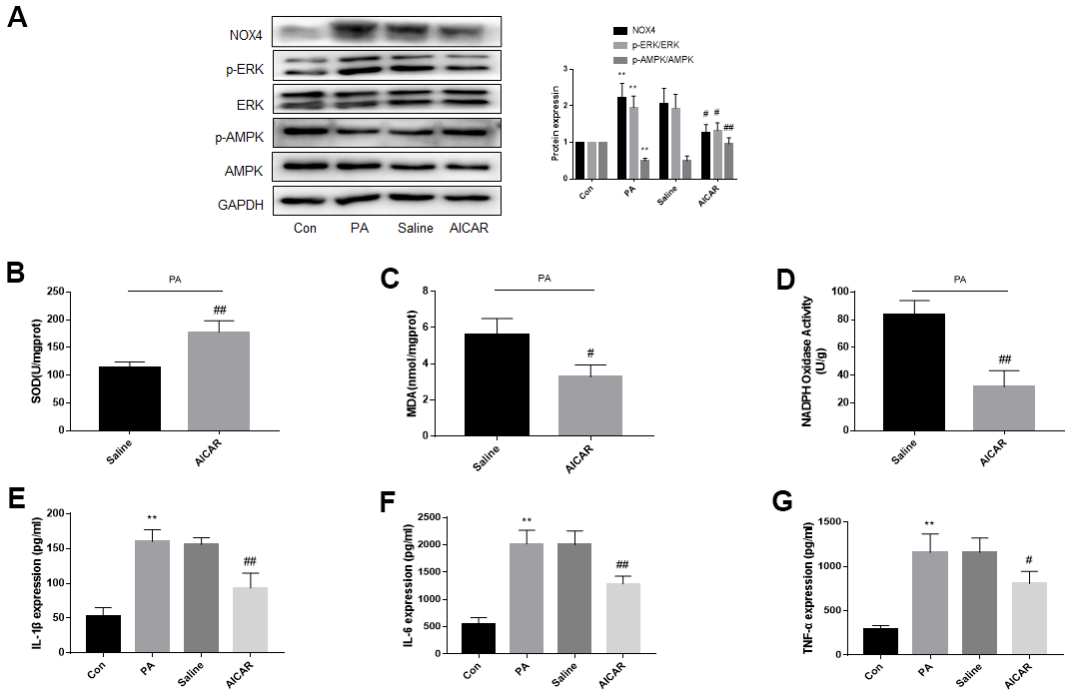
**AMPK activation reduces ERK phosphorylation, NOX4 expression and improves oxidative stress and inflammation of DRG neurons cultured in PA-added medium *in vitro*.** DRG neurons isolated from rats were cultured. PA was applied to simulate a high-fat environment and AICAR to activate AMPK. (**A**) The expression of p-AMPK, AMPK, p-ERK, ERK, and NOX4 in DRG neurons were detected by western blot. (**B**–**D**) The supernatant of cultured cells was collected and SOD, MDA, NADPH oxidase activity in the supernatant were detected by kit. (**E**–**G**) The supernatant of cultured cells was collected and the secretion of IL-1β, IL-6, and TNF-α was measured by ELISA. N = 5 per group, **P < 0.01 vs. con group, ^#^*P* < 0.05 vs. saline group, ^##^*P* < 0.01 vs. saline group. AMPK, AMP-activated protein kinase; ERK, extracellular-regulated kinase; NOX4, NAD(P)H oxidase 4; DRG, dorsal root ganglia; PA, palmitic acid; AICAR, AMPK agonists; MDA, malondialdehyde; SOD, superoxide dismutase; IL-1β, interleukin-1 beta; IL-6, interleukin-6; TNF-α, tumor necrosis factor alpha; ELISA, enzyme-linked immunosorbent assay.

We stimulated DRG neurons with AICAR to activate AMPK. Similar to the previous *in vivo* experiments, the AMPK phosphorylation decreased, and the ERK phosphorylation and NOX4 expression increased in DRG neurons after PA stimulation. AICAR treatment, but not saline treatment, increased AMPK phosphorylation, and decreased ERK phosphorylation and NOX4 expression (Figure 7A).

Figure 7B–7G showed that PA increased inflammation and oxidative stress in DRG neurons. Inflammation and oxidative stress were more severe in the AICAR-treated group than the saline-treated group.

All these data in Figures 6, 7 suggest that the abnormal inactivation of AMPK may lead to the injury of nervous system through ERK-NOX4 pathway, which may lead to an increase of neuropathic pain in obese rats.

## DISCUSSION

AMPK-ERK-NOX4 pathway may contribute to the increased neuropathic pain in obese rats is the major finding of this work. This view is based on the following evidences: (1) Under high-fat environment, the neuropathic pain of obese rats, the expression of p-ERK and NOX4 in nervous tissue increased, accompanied by increased inflammation, oxidative stress and apoptosis. Inhibition of ERK activation significantly improved the above pathological conditions. (2) Rescue experiments showed that overexpression of NOX4 abolished the anti-oxidative stress, anti-inflammation, and anti-apoptotic effects produced by an ERK inhibitor. (3) High-fat decreased AMPK phosphorylation, and promoting AMPK activation inhibited the ERK-NOX4 pathway and was accompanied by a reduction in neuropathic pain, as well as decreased oxidative stress and inflammatory response in nervous tissue.

Neuropathic pain caused by obesity has become a serious public health problem. There are not enough studies on obesity-induced neuropathic pain, and therefore the precise mechanism is still not fully elucidated, and the effective clinical treatments are lacking. Our previous studies have confirmed that MAPK/ERK contributes to CCD-induced neuropathic pain, and inhibition of ERK phosphorylation with U0126 and interference of ERK expression with shRNA can alleviate pain [8, 9]. Combined with previous studies showing that in smooth muscle cells and adipocytes, treatment with lipids can lead to ERK activation [10, 11], we hypothesized that the elevated neuropathic pain in obese rats might be related to the abnormal activation of ERK in nervous tissue. As expected, ERK phosphorylation in spinal cord and DRG tissues of obese CCD rats increased compared with normal CCD rats, and after intrathecal injection of U0126, ERK phosphorylation was inhibited, accompanied by decreased neuropathic pain in obese CCD rats. This result confirms our conjecture. *In vitro* and *in vivo*, nervous tissue exhibited elevated inflammation, oxidative stress and apoptosis under high-fat environment, but these pathological conditions improved after inhibiting ERK activation. Since inflammation, oxidative stress and apoptosis has a close relationship with neuropathic pain [24–31], increased hyperalgesia in obese rats may be induced by the above pathological mechanism, and ERK inactivation may improve pain in obese rats by inhibiting inflammatory, oxidative stress and apoptosis of nervous tissue.

Next, we explored the molecular mechanism by which ERK phosphorylation elevates neuropathic pain. Elevated NOX4 contributes to neuropathic pain [32, 33] and leads to inflammatory response [18, 34–36]. So, we detected the levels of NOX4 in the nervous tissue of obese CCD rats, and investigated the change in NOX4 expression after inhibiting the activity of ERK by western blot *in vivo* and *in vitro*. NOX4 protein levels in the nervous tissue of obese CCD rats was higher compared with normal CCD rats, but the expression of NOX4 was significantly decreased after inhibiting ERK activation in the nervous tissue of obese CCD rats. The *in vitro* findings were consistent with the *in vivo* results. Furthermore, after NOX4 overexpression, the anti-oxidative stress, inflammatory and apoptotic role of an ERK inhibitor on DRG neurons under high-fat environment were abolished. Based on this, we conclude that the ERK phosphorylation induced increase of neuropathic pain in obese rats may be NOX4-dependent.

As an important molecule, AMPK plays a pivotal role in the moderation of bioenergy metabolism and has an important effect on diabetes and other metabolic diseases. As previously reported, AMPK is associated with inflammatory pain, incision-evoked pain, and CCI-induced pain [20–23]. We explored whether the inactivation of AMPK in the nervous tissue of obese rats was responsible for the ERK-NOX4 pathway activation, which may result in an increase of neuropathic pain. AMPK phosphorylation in nervous tissue and DRG neurons decreased in a high-fat environment *In vivo* and *in vitro*. After AMPK activation, ERK phosphorylation and NOX4 expression decreased, and the inflammatory response, oxidative stress and neuropathic pain were also alleviated. From these results, we conclude that AMPK is inactivated in the nervous tissue of obese rats, which possibly leads to increased inflammation, oxidative stress in the nervous tissue and increased neuropathic pain in rats by activating the ERK-NOX4 pathway.

**Figure 8:**
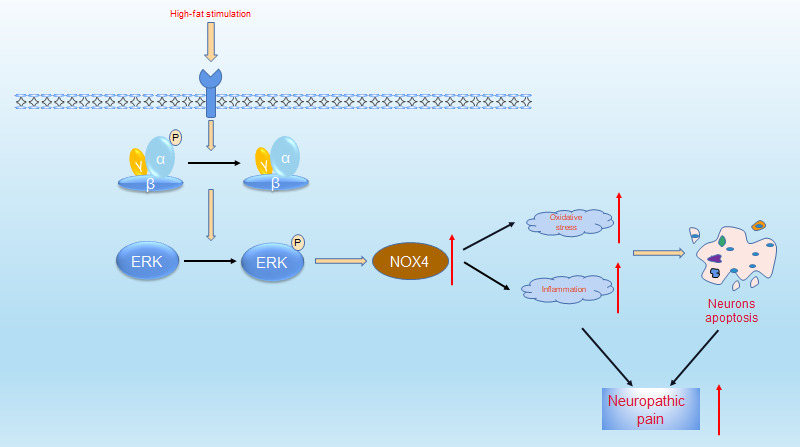
**Proposed mechanisms by which obesity leads to increased neuropathic pain.** Under high-fat environment, AMPK is inactivated in neuronal cells, then ERK is activated, thus the expression of NOX4 is elevated. These changes elevate intracellular levels of oxidative stress and inflammation, which ultimately leads to neuronal apoptosis. These pathologies resulted in increased neuropathic pain.

In summary, as shown in Figure 8, the increased neuropathic pain in obese rats may be due to abnormal alterations in the AMPK-ERK-NOX4 pathway. In the future, targeting the AMPK-ERK-NOX4 pathway may be an effective treatment strategy for hyperalgesia in obese patients.
